# Where do we invest money to implement active learning on caries detection? An economic evaluation

**DOI:** 10.1590/1807-3107bor-2024.vol38.0055

**Published:** 2024-06-24

**Authors:** Jhandira Daibelis Yampa VARGAS, Thais Gomes MACHADO, Gabriele OLIVEIRA, José Carlos Pettorossi IMPARATO, Daniela Prócida RAGGIO, Fausto Medeiros MENDES, Mariana Minatel BRAGA

**Affiliations:** (a) Universidade de São Paulo – USP, School of Dentristry, Department of Orthodontics and Pediatric Dentistry, São Paulo, SP, Brazil.

**Keywords:** Costs and Cost Analysis, Resource Allocation, Teaching, Health Education, Dental, Students, Dental

## Abstract

This trial-based economic evaluation aimed to estimate the incremental cost of implementing an active learning strategy (theoretical-practical workshop) to substitute the didactic lecture as the sole method for students training in caries detection. We also provided a budget impact analysis and explored the composition of costs related to the activity. Data from the coordinating centre of a multicentre randomized and controlled study (IuSTC01) was analyzed as the first part of our main economic analysis plan. The perspective of the educational provider (the institution implementing the activity) was considered, and an immediate time horizon was adopted. All used resources were valued in Brazilian Real by adopting a microcosting strategy. Costs for each strategy were estimated and converted into international dollars. The incremental Cost per student and the total cost of implementing the complete teaching strategy for 80 students were calculated. Monte Carlo simulations were used to estimate the uncertainties. The incremental Cost estimated for the workshop implementation would be $7.93 per student (interquartile range (IQR): $7.8–8.1), and the total cost of the teaching activity would be $684 (IQR:672–696). The laboratory training comprised more than 50% of the total amount spent, and a higher percentage of this value was related to human resources costs (72%). Saving 40% of the costs could be expected for the next rounds of activities in the institution, assuming no need for additional preparation of didactic materials and tutor training. A modest incremental cost per student and an acceptable organizational budget impact should be expected for the institution when including active learning training in caries detection for undergraduate students, mainly related to the human resources involved.

## Introduction

Active learning is an instructional approach that places greater responsibility on the learners, by giving them more control over their own learning process and increasing their motivation and engagement.^1^ This student-centred approach promotes a deeper understanding of contents^2^ and has been related to positive outcomes in health education.^3^ Besides, students have an optimistic perspective of it.^4^ Such benefits have motivated the amendment of many higher education programs, including for health professionals,^5,6^ by adding these strategies to the curriculum. For this audience, using active learning is important as it promotes the development of critical thinking and problem-solving skills, considered essential attributes for a well-trained healthcare team.^7^.

Besides individual challenges for teachers and learners, the demand for human and technological resources, budget limitations, or insufficient time and support provided by educational institutions may be obstacles for implementing a new active learning method.^8^ In some cases, the lack of resources can be a crucial factor for not adopting active-learning instructional approaches in the program, even making them impracticable in different contexts.^9^

Little attention has been paid to the economic impact of educational strategies on universities and society. Nevertheless, different educational strategies require different resources and, consequently, different “costs” (sometimes extra costs).^10^ Active learning strategies may require extra time, additional staff, and other resources. Therefore, estimating the costs expected or spent on an educational activity like this seems imperative to the implementation process. Economic evaluations are performed in education to prove whether allocating more resources effectively justifies introducing new pedagogical methodologies. However, in the literature, most cost studies in dental education are related to the student’s debts, rather than the cost of acquiring skills or getting good professional training.

In the Pediatric Dentistry program at the University of São Paulo, a 10-year active learning educational strategy has been successfully implemented for training undergraduate students in caries detection.^11^ It was implemented in 2009 to substitute the conventional passive learning strategy (didactic lectures as the sole method) as part of a wider initiative (Initiatives for undergraduate Students’ Training in Cariology – IuSTC). It has promising effects on students’ performance.^12^ This method uses teaching systems such as preclinical simulation, think-pair-share, small-group discussions, and prompt personalized tutor feedback, as described in previous publications^11^. This pedagogical strategy has served as an example for other dental schools that also began to apply it with their students, motivating a multicentre trial^13^ to explore their educational effects. Besides, the economic aspects of such activity implementation (and its continuity) remain unknown. That is why the multicentre study has aimed to explore such aspects, as well as the educational ones.^13^

In that sense, the first attempt was to investigate how the resources are allocated to implement the activity in ideal conditions. We considered the coordinating centre as the centre that provides the ideal conditions we require for such analyses. Therefore, the present work aimed to perform a cost analysis to explore the resources allocated to implement an innovative active learning strategy to teach caries detection to undergraduate dental students and estimate the immediate additional cost spent, instead of only using the conventional strategy: a didactic lecture.

We believe this pioneering study can highlight the importance of strategic resource allocation in dental education and may establish some parameters for future studies linking economy and education. By exploring the cost composition related to different educational strategies and revealing a budget impact analysis for the provider educational institution, it is possible to understand the economic impact of introducing active learning in such a context.

## Methodology

This article presents a partial economic educational evaluation nested in a randomized and controlled interventional study on dental education. The study is designed to compare the theoretical-practical workshop to the traditional didactic lecture as the sole strategy for training undergraduate students in caries detection. Since we were training future healthcare professionals, we assumed the educational intervention to be a health technology and used the CHEERS checklist to guide our report.

This study reports findings from the first phase planned in the main educational economic analysis plan (www.osf.io/wa6x2),^14^ focusing on exploring the cost composition and budget impact of the proposed educational strategy in ideal circumstances. The coordinator’s reality was assumed to provide such an ideal scenario. Therefore, data were gathered in the coordinating centre of a multicentre randomized and controlled study (IuSTC01).^13^ This centre was the precursor in implementing the activity and was used as reference for the cost analysis.

**Figure 1:**
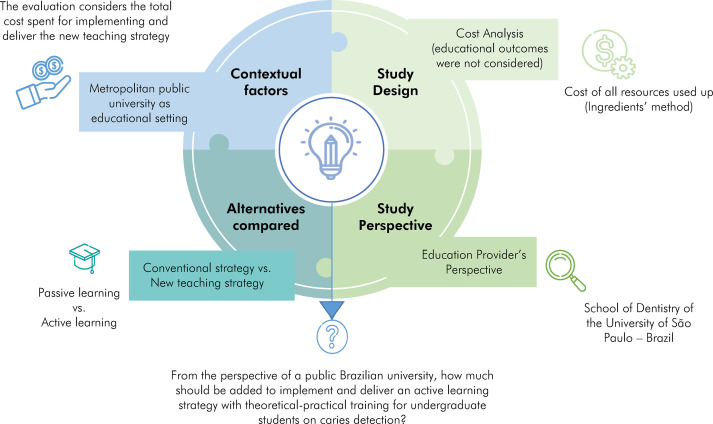
Guided structure for cost analysis in Health Professionals Training.

Figure 1 presents the study framework and the focused economic research question. This evaluation considered the perspective of the educational provider for the cost assessment analysis and an immediate time horizon (single educational intervention).

### Registration and ethical considerations

The multicentre study protocol was approved by the Ethics in Research Committee (CAAE 39632614.0.0000.0075). The student’s performance in scoring caries lesions was assessed immediately after they completed the entire pedagogical activity; however, those results were not considered for this analysis and will be considered in future publications of the main protocol. The original protocol was already published and is considered the official registration of the IuSTC01 study.^13^ In addition, to ensure the transparency of the economic evaluations, the educational economic analysis plan was made available in an open repository (www.osf.io/wa6x2).^14^

### Study setting and population

The active learning strategy under evaluation was implemented by Pediatric Dentistry lecturers in 2009 at the University of São Paulo, Brazil (FOUSP).^11,13^ Since then, the activity has been carried out with its undergraduate students every year in the full-time and evening courses. For this economic evaluation, a class with 80 students enrolled in the Pediatric Dentistry course that year was considered. This is the average annual enrollment in the full-time course, which was assumed as our base case.

### Interventions

This cost evaluation included two interventions to teach caries detection to undergraduate dental students to verify our research question: 1. conventional educational strategy (didactic lectures) versus 2. alternative strategy (didactic lectures associated with the active learning strategy using guided tutored feedback).

**Figure 2:**
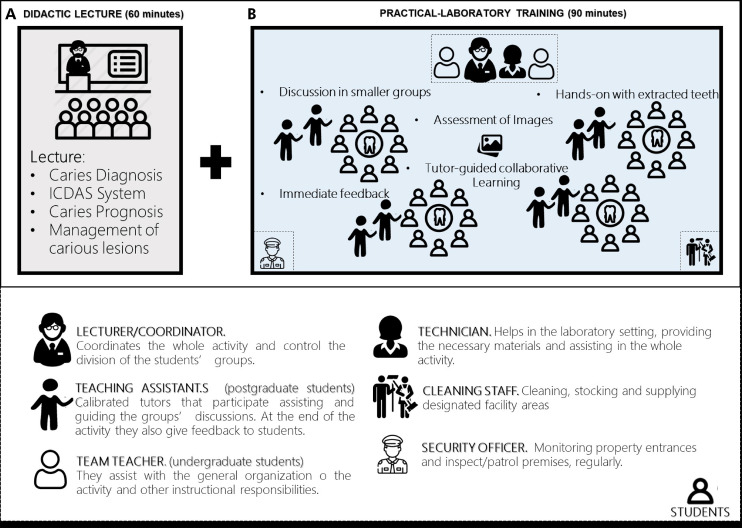
Schematic representation of the active learning activity (A+B), different actors involved, and their responsibilities/assignments during the activity.

Didactic lecture: a conventional lecture on caries detection was given by one of the Pediatric Dentistry faculty professors. She applied the resources normally used for that and audiovisual materials prepared throughout her career for such educational purposes. The lecture lasted approximately 60 minutes (Figure 2A).

Didactic lecture associated with active learning strategy: the complete activity combined the didactic lecture, as described above, and the active learning strategies (Figure 2 A, B). For the active learning strategy (Figure 2B), students were divided into small groups tutored by graduate students (MSc or PhD candidates in Pediatric Dentistry). This preclinical activity is performed before students interact with real patients at the pediatric dental clinic. They were exposed to different clinical situations in images and extracted teeth, for which they are supposed to detect caries lesions and make assessment decisions. Discussions were raised from each clinical situation based on students’ answers, not the tutor’s correct answers, allowing for a thinking-pair approach. Tutors were responsible for guiding the discussions and ensuring that the intentional content had been appropriately explored.

A) Details about the didactic lecture. B) Details about the activity-learning phase of the entire activity.

**Figure 3:**
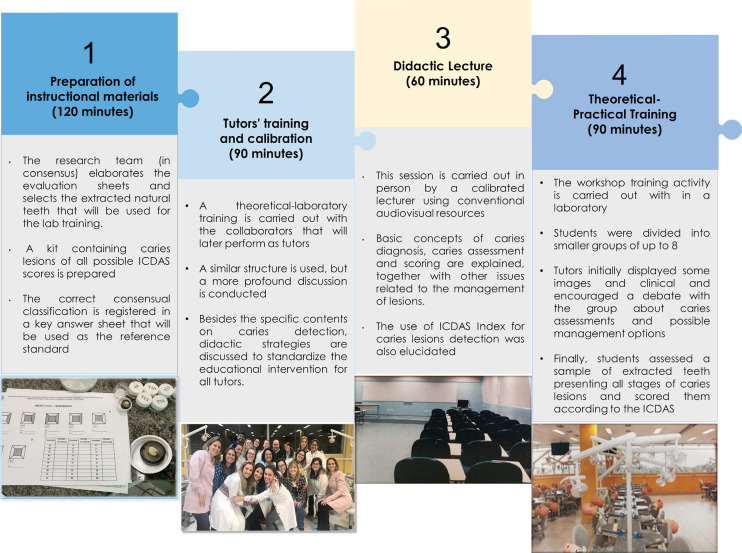
Tutored theoretical-practical learning activity for training in caries detection – phases and components.

This new active learning activity was accomplished with the participation of many collaborators (Figure 2) and consisted of four main phases, as displayed in Figure 3. Phases 1 and 2 preceded the effective application, while phases 3 and 4 correspond to the educational intervention with the students (Figure 3).

Phases: 1 - kit of extracted teeth prepared for the workshop training phase, 2 – a group of trained tutors, 3 - University facilities used for didactic lectures, 4 - University facilities used for practical-laboratory training).

### Cost estimation method

A micro-costing approach was used to estimate the cost of implementing the proposed active learning activity. In this approach, we enumerate and assign value to every input for the pedagogical activity. Furthermore, the “ingredients method”^15^ was also adopted because it is commonly used in economic research and facilitates economic evaluations in the educational context.^15^ It involves identifying and specifying all the “ingredients” needed for the educational activity and determining the monetary value corresponding to each ingredient.

**Figure 4:**
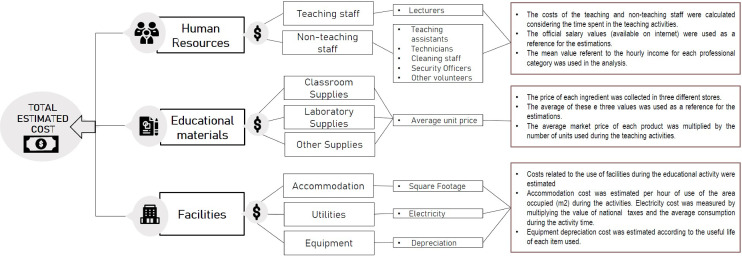
Final cost composition parameters.

The ingredients, i.e. all the resources used, were organized according to the implementation phases (Figure 3). Only phase 3 of Figure 3 was valued for the didactic lecture, while for the combination of didactic lecture and active learning strategies, phases 1 to 4 from the same figure were considered. The resources were classified into three main categories for all phases: human, material, and facilities (Figure 4). Cost valuation was determined according to Figure 4 assumptions. For the staffing cost, only the hours worked were considered based on the activity duration, since it was impossible to estimate the value of the preparation of each educational staff appropriately. To minimize this gap, we consider professional history according to their level of education, taking their development throughout their careers as a basis for the time they spent preparing for their respective role in such activities.

The final composition of costs was obtained from the sum of all these categories. Prices were collected in Brazilian Real (BRL) in 2021 and then converted into International Dollars ($) using Brazil’s purchasing power parities (PPPs) in 2021 (Exchange rate: 2.53). For this first-phase analysis, cost adjustments were unnecessary because we assumed the immediate educational intervention as a one-off event. Therefore, it was not necessary to consider price variations over time, such as inflation or discount values.

### Data analysis

As defined in Figure 2, the costs for each intervention were estimated. Then, the incremental cost per student for implementing the laboratory activity that complements the didactic lecture was estimated. The subtotals of each of the four phases described were considered for the active-learning activity to illustrate the cost composition of the different stages of implementation. The total cost for implementing the complete strategy for 80 students (organizational budget impact) was also calculated.

Monte Carlo simulations were used to estimate the uncertainties. For that, we assumed a triangular distribution of subtotal costs of each stage of the activities. The value obtained in the reference sample was used as the mean of the distribution. Uncertainties were computed with varying costs ± 10%, simulating other possible contexts. Then, the interquartile range (IQR) was calculated to estimate the uncertainty in the values calculated.

## Results

The total time to implement this active learning strategy for teaching cariology was approximately 6.5 hours, while for the didactic lecture, approximately one hour was spent. For delivering the activity to a class of 80 students, 25 collaborators implemented and effectively applied the pre-specified educational approach. Students were divided into 8 different groups and performed the activity in one single round.

The total cost to implement and deliver an educational strategy for training 80 undergraduate students in caries detection was $684 (approximately $8.5 per student) (Table 1). An incremental cost of $8 per student for the workshop implementation for substituting the didactic lecture was generated.

**Table 1:** Cost composition of educational activities analyzed in this economic evaluation and its interquartile range (IQR) obtained with Monte Carlo simulation models – median (IQR).

Stages	Duration time	Cost of human resources ($)	Cost of educational material ($)	Cost of facilities ($)	Median cost per stage (IQR)	Percentage of cost per stage(%)
Lecture + Practical laboratory training						
Preparation of instructional material	1 hour class	79.83	0	16.03	**95.86 (93.05 - 98.66)**	14.02
Tutors training and engagement	1.5 hour class	153.52	0	37.35	**190.87 (184.72 – 195.87)**	27.92
Didactic lecture	1 hour class	38.02	3.05	08.03	**49.10 (48.25 – 51.16)**	7.18
Practical laboratory training	1.5 hour class	218.99	104.29	24.43	**347.72 (93.05 - 98.66)**	50.86
Total Median Cost (IQR)		**490.37** (107.60–111.37)	**107.34** (107.60–111.37)	**85.85** (80.70–83.52)	**683.50 (672.49 – 696.07)**	**100**
% Cost composition		72	16	12		**100**
Complete educational strategy (Implementation and delivering)	**6 hours**	**$ 683.50 (672.49–696.07)**				
Cost per estudent	-	**$ 8.55 (8.40–8.70)**				
Didactic lecture						
Only Lecture	1 hour class	38.01	7.72	8.03		100
% Cost composition		78	6	16		**100**
Total Median Cost (IQR)		**$ 49.10 (48.25–51.16)**				
Cost per Student		**$ 0.62 (0.60–0.64)**				
Incremental Calculations						
Incremental Total Cost		**$ 634.46 (622.88–646.33)**				
Incremental Cost per student		**$ 7.93 (7.79 –8.08)**				

Highlighted cells may be phases that could be reduced or extinguished in case of successive activities.

Phase 4 (laboratory training) represented the largest part of the activity cost, constituting about 50% of the total cost (Table 1). When we analyze the cost composition, more than 70% of the money was spent on human resources. Staff participation and its cost were expressed in Table 2, showing how the values were distributed among staff members for different tasks performed during the active learning activity (Table 2, Figure 2). Other minor cost components were divided equally between the remaining expenses in implementing the activity (Table 1).

**Table 2:** Cost of staff participation and tasks performed during the active learning activity.

Implementation phase	Number of people involved	Description	Number of people involved	Average cost per hour (R$)	Average cost per hour (PPPs)
Preparation of instructional material	3	Coordinator	1	86.22	34.08
		Assistant Teacher	1	12.50	4.94
		TEAM* teacher	1	2.27	0.90
Tutors’ training and engagement	19	Coordinator	1	86.22	34.08
		Tutors	16	10.51	4.15
		TEAM* teacher	2	2.27	0.90
Didactic Lecture	1	Lecturer	1	96.18	38.02
Practical laboratory training	25	Coordinator	1	86.22	34.08
		Assistant Teacher / Tutors	16	10.51	4.15
		TEAM* teacher	2	2.27	0.90
		Lab technician	1	49.53	19.58
		Cleaning staff	4	27.56	10.89
		Security officer	1	6.25	2.47

*TEAM refers to Teacher Education And Mentoring (TEAM) Program.

For future applications, we could predict savings of up to 40% of the total cost if we consider the phases of preparing didactic materials and training tutors (assuming no substitutions would be necessary for future occasions) may be unnecessary.

## Discussion

Implementing active learning strategies may represent an effort to transform education by improving teaching methods, and it should also be considered an economic investment in education. In our findings, we highlighted that an educational institution should provide an extra budget or an incremental investment of approximately 8 dollars per student to provide an innovative active learning strategy for caries detection training for undergraduate students, adding up to approximately 700 dollars to deliver this activity to a whole class of 80 undergraduate students. To interpret those findings, it is certainly relevant to consider the institutional investment in education. The University of São Paulo, for example, in 2016, invested $2,065^1^ monthly per student enrolled in faculties with complex infrastructure (laboratories and clinics),^16^ such as its dental schools, equivalent to approximately $2,500^2^ nowadays. In such context, we are inclined to consider the resources invested in implementing such additional educational activity as a modest investment.

Decision-making thresholds are usually an important concern in economic evaluations.^17^ Especially for education, this issue is even more complex compared to other interventions in Health. The perception of such “value” depends on many factors, and, in this case, it is directly related to the stakeholders’ interests. In this sense, other student-centred parameters may also be interpreted, for instance, the relation between quality, price, and ‘gross benefit perceived by the user^18^. Such additional views may make the investment of 8 dollars per student (monetary value) seem inexpensive or expensive, depending on the context and the learning effect (educational benefit) the teaching method could bring to the students.

On the other hand, we focused this study on a cost evaluation of the proposed educational alternative and not specifically on its effects. Such analysis was planned beforehand in our economic analysis plan and is part of a broader multicentre study that has different objectives.^13^ The idea was to explore the ideal conditions of the coordinating centre, where the strategy was created to explore resource allocation and consequent cost composition. The exclusive publication of these results can focus on disseminating the methodology and these interesting findings. Moreover, this paper can be an important guide for other cost evaluations of educational strategies in Dentistry and other Health areas. Even though it is not part of this work, it is well known that active learning has been widely recommended when educating health professionals such as dentists, because it increases student engagement, promotes peer collaboration, enhances reflection and helps develop better reasoning and critical thinking skills, in addition to problem-solving abilities^3,19^ and general cognitive processes^20^. The educational effects of the active learning strategy used for caries detection have been *de facto* previously explored and published.^12^

Preparing for any activity demands time and effort. Even the didactic lecture preparation is time-consuming. However, it was challenging to assign a monetary value to that since it could represent the combination of a lecturer’s lifelong preparation during their academic career and the result of the accumulated knowledge and expertise acquired over many years. That is why we assumed no additional cost for lecture preparation, and it did not pose a problem for such evaluation since it was included in both interventions. We consider professional time according to their level of education and training, assuming their development throughout their career as an indicator of the time they spent preparing for their respective role in all performed activities. Furthermore, variations around this value could also be explored when sensitivity analyses were performed and cost uncertainties were estimated.

The preparation for active learning strategies may be more time-consuming and expensive than the delivery phase itself.^21^ Pathways to guide the activity should be prepared to ensure all class objectives can be reached at the end, even if different approaches are chosen during the activity, since a student-centred approach is being used. This aspect should be even more relevant if we consider that a team of professionals will be required to deliver the activity, as discussed so far. The extra time and effort to implement this strategy demand an incremental cost usually expected and observed in other active learning strategies.^21-24^

An important finding emerging from this study is the magnitude of the human resources invested in such activity, which fundamentally used tutored guided feedback for undergraduate students. Over 70% of the total amount was invested in the task force to implement the activity. Additional time dedicated from staff is related to the most expensive component when implementing student-centred teaching strategies^22^.

Several tutors should have been involved in the teaching activities to achieve the training goals in such laboratory activities. Smaller groups of students guided by one or two tutors ensure that the active learning foundation (such as thin-pair share or collaborative learning and immediate feedback) were kept. The proper conduction of discussions in the smaller groups was essential and would be impossible in a large group of students (a group of 80 students, for example). Therefore, the more tutors we can have participating, the more individualized the teaching process becomes, and it enables students to effectively participate in the discussions, receiving immediate feedback for their questions.^25^ In smaller groups, the undergraduate students may feel more comfortable and have more opportunities to actively participate compared to when they are in a regular classroom with all students together.

Specifically for this educational activity, the teaching staff was mainly composed of graduate students in the coordinating centre where the activity was created, implemented and explored for such analysis. They acted as assistant professors (as part of their academic training). Often, these graduate students only receive a grant for studying at the university. That is why, in this context, this strategy may save financial resources when this type of activity is implemented. Implementing an activity involving regular professors can be much more expensive and sometimes deterrent, given the labour-intensive work involved.

On the other hand, alternative strategies may be found to minimize these issues and will be published elsewhere due to IuSTC initiatives.^26^ These possible differences were mathematically considered using sensitivity analyses in the present economic evaluation. They will be explored deeply in future evaluations planned for this multicentre trial, exploring different scenarios and contexts.

Savings of approximately 40% can be expected on future occasions if the activity is delivered by the same group of staff every year since retraining the teaching staff or replacing the teaching materials would not be necessary. In fact, it has been shown that for some active online learning strategies for continuing medical education, the initial costs for planning, developing, and implementing tend to be amortized in subsequent offers.^27^

The active learning strategy proposed here to train undergraduate students in caries detection requires, by its nature, a different educational environment compared to a regular lecture. Despite not being the most expressive cost component, the structural costs are approximately 10 times greater than the traditional teaching approach (the didactic lecture). Simple laboratories would be enough to carry out the described activity, and very expensive equipment or “luxury” facilities are unnecessary. Possibly, the infrastructure of each institution will be adapted for use, and minimal extra costs should be expected. However, this economic analysis valued and considered the usage time of such an environment (rather than a specific supply or more advanced technologies).

The teaching materials were provided to the students in this study. It is possible to say that the most important element for the practical caries detection simulation experience was the sample of extracted teeth prepared for this activity. We must understand that preparing some instructional material during the implementation phase will normally require an upfront cost. However, this cost will be gradually lowered in future applications, as they will have already been developed. Exceptions are cases where a sample tooth or other specific material needs to be replaced due to any damage or deterioration caused by usage. Therefore, there will be a new associated cost, but it will be smaller and will not affect the overall cost of the activity. On the other hand, should we need to prepare a new complete set of teeth, the cost would probably be higher as personnel and material would be required to prepare those sets of samples.

The teaching staff training phase is another important aspect that cannot be overlooked as it is an important input for economic evaluation. In theory, several issues may affect the teaching activity if the staff is not well aligned and engaged during the team formation process^28^. The formation of the team involves several stages, and it is essential to understand whether these collaborators’ inputs and outputs can modify the dynamic of the teaching activity^28^, which reinforces the relevance of “spending” time and funds in training collaborators who will be involved in implementing a teaching activity. Working as a functional team poses a great challenge, but this factor cannot influence the success of the group and the teaching-learning activity.

Applying student-centered teaching approaches can transform education. In Cariology, lectures seem to be the most used method of teaching students. Nevertheless, as we demonstrated in this paper, it would be possible to introduce new teaching strategies without excessive costs. However, implementing new active teaching strategies can often be challenging for some institutions as they have been described as more expensive and time-consuming, discouraging their implementation^9,29,30^. We believe this paper can contribute to this area of research by demonstrating the required resources to implement and deliver active learning strategies, as described here. It can also start a new trend in studies. Although focused on the Brazilian perspective, we used Monte Carlo simulations considering a distribution of different values around the registered central value. This way, we believe other national and international contexts were represented.

So far, most studies consider costs in dental education only related to students’ debts,^31^ not costs for acquiring skills or obtaining good professional training. Cost disclosure in educational research is still infrequent and incomplete.^30,32^ This issue has probably received limited attention in the literature due to the difficulty in assigning monetary values to a set of teaching activities or the entire educational process.^15^ However, a recent publication has reinforced the importance of exploring and reviewing the concepts of “costs” and “values” in the education of health professionals.^33^ The results of this study, detailing and estimating the costs of a teaching method, could be used as a starting point for future comparisons with other studies and other centres that used the same teaching methodology. Further research is still needed to understand the economic and non-economic implications that may influence the efficiency of the teaching methods. Awareness about this topic is important for allocating economic resources and improving the quality of education.

## Conclusion

A modest incremental cost per student and an acceptable organizational budget impact should be expected by the institution when including active learning training in caries detection for undergraduate students. This extra cost is mainly related to the human resources involved in planning, preparing, and delivering these activities.

## Footnotes

1 Conversion rate in 2016 $1 = BR$2.12, according to OECD data (https://data.oecd.org/conversion/purchasing-power-parities-ppp.htm).

2 Conversion rate in 2012 $1 = BR$2.53, according to OECD data (https://data.oecd.org/conversion/purchasing-power-parities-ppp.htm).
